# Prediction of Cascading Failures in Spatial Networks

**DOI:** 10.1371/journal.pone.0153904

**Published:** 2016-04-19

**Authors:** Yang Shunkun, Zhang Jiaquan, Lu Dan

**Affiliations:** School of Reliability and Systems Engineering, Beihang University, Beijing, China; University of Texas at Austin, UNITED STATES

## Abstract

Cascading overload failures are widely found in large-scale parallel systems and remain a major threat to system reliability; therefore, they are of great concern to maintainers and managers of different systems. Accurate cascading failure prediction can provide useful information to help control networks. However, for a large, gradually growing network with increasing complexity, it is often impractical to explore the behavior of a single node from the perspective of failure propagation. Fortunately, overload failures that propagate through a network exhibit certain spatial-temporal correlations, which allows the study of a group of nodes that share common spatial and temporal characteristics. Therefore, in this study, we seek to predict the failure rates of nodes in a given group using machine-learning methods.

We simulated overload failure propagations in a weighted lattice network that start with a center attack and predicted the failure percentages of different groups of nodes that are separated by a given distance. The experimental results of a feedforward neural network (FNN), a recurrent neural network (RNN) and support vector regression (SVR) all show that these different models can accurately predict the similar behavior of nodes in a given group during cascading overload propagation.

## Introduction

Cascading failures in complex networks negatively affect system reliability. Several studies have investigated possible laws that govern the cascading failure process using percolation theory [1,2]. However, improving the reliability of complex systems requires further research [3]. Researchers have increasingly used failure prediction and reliability prediction in reliability engineering. The majority of previous studies have focused on a single system, such as software, railways, or a large system containing several similar components [4–7]. However, the prediction of cascading failures in networks remains underdeveloped. Because reliability and failure have a strong correlation with time, most previous research made predictions based on time series. In spatial networks, such as transportation networks and power grids, cascading overload failures typically start from local components that exhibit not only temporal but also visible spatial correlations [8–10]. Few studies have attempted to predict cascading failures in networks related to spatial characteristics. Zhao et al. found that cascading overload failures propagate at a constant velocity in spatially embedded networks. This meaningful discovery provides an efficient method of characterizing the dynamics of an entire network. However, the velocity of a cascading overload failure is a statistical parameter that cannot provide detailed information about the behavior of the nodes. Thus, a constant velocity provides the possibility of predicting failures based on spatial characteristics. In this study, we studied different groups of nodes that are separated by different spatial characteristics with regard to failure prediction instead of focusing on a single node of a network or on an entire network at different times.

To satisfy the larger amount of users and meet the rapidly increasing complexity of current system function, most large-scale systems contain subsystems that can complete simpler tasks by themselves. However, as the load increases, a single subsystem cannot manage all of the tasks and moves a certain amount of its load to its neighbors such that the entire system can operate steadily and efficiently. A problem can developed in such a combined system leads where the load will be released to other components if certain components break down due to attack or random failure. When a subsystem’s load exceeds a given threshold, it will break down due to overload; then, the entire system begins to degrade in sequence, producing a cascading overload failure [11,12]. In certain cases, subsystems cannot be repaired after an overload that causes a failure, which may lead to unnecessary financial loss. Accurate predictions of the cascading failure process could prevent certain subsystems from undergoing overload failure and reduce this unnecessary loss.

Conversely, when maintaining a relatively large-scale system, the complexity of the network and the limited resources make it impossible to manage each node individually [13]. It is often unnecessary to focus on whether one single subsystem will overload when the entire system is suffering from failure propagation. Instead, focusing on a group of nodes that share the same characteristics can make the task more efficient. Considering the limitation of maintenance resources, it is important to determine how to efficiently allocate resources (e.g., people, materials) to stop or slow the failure’s propagation as quickly as possible. For example, when certain nodes in a power grid fail, it is difficult or impossible to determine which one of the remaining transformers is going to overload due to the subsequent load redistribution. However, the probability that a node tends to break down can be determined. Then, loads can be manually delivered to the nodes that are the least likely to overload. We can arrange different percentages of the limited resources to different nodes based on accurately predicted data.

## Materials and Methods

### Cascading overload failures

**Fig 1:**
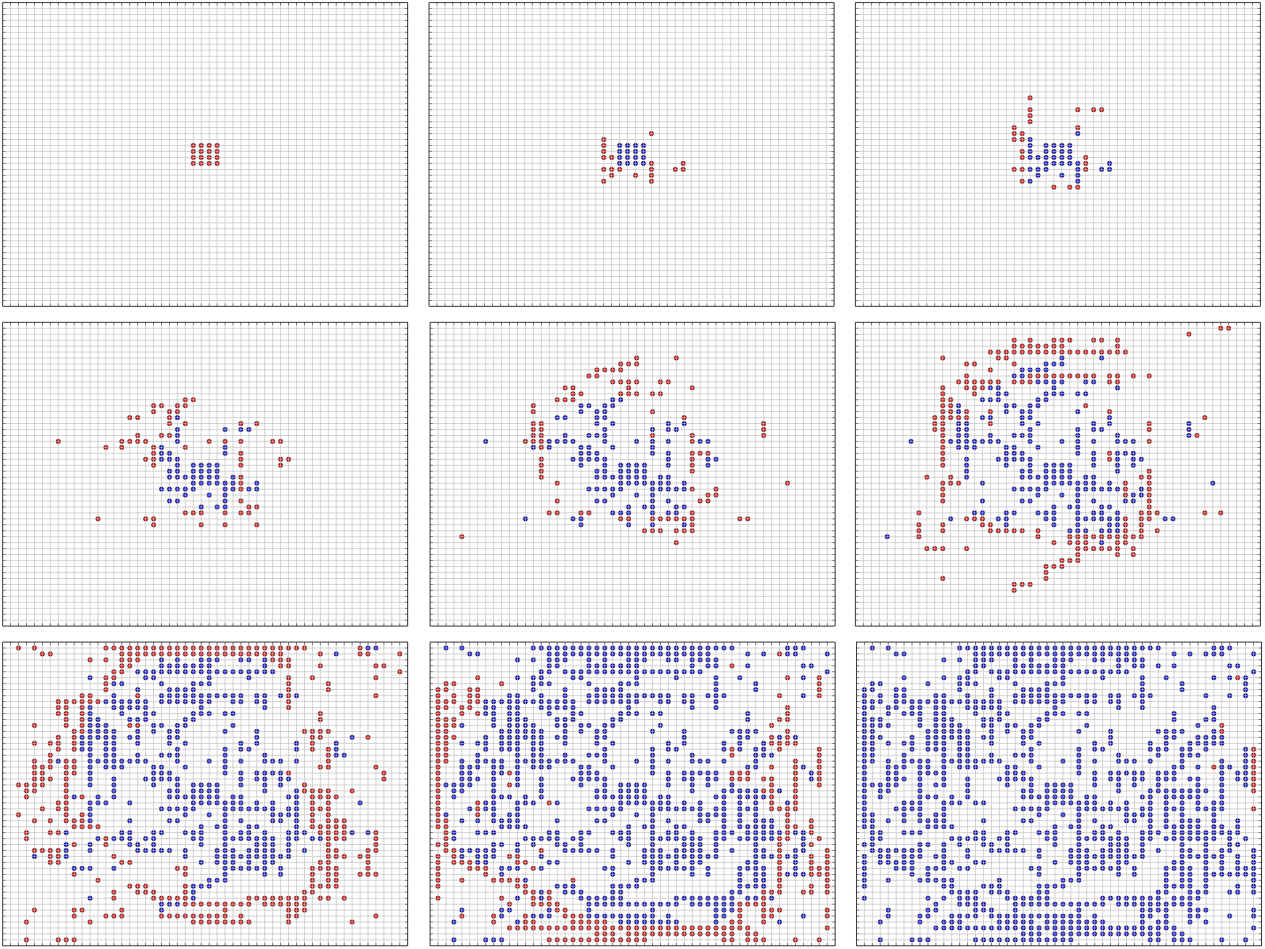
Cascading overload failures with α = 1.0. The red nodes are new failures in the current step; the blue nodes are failures that occurred before the current step.

Networks, such as transportation networks and power grids, are similar to lattice networks on a macro scale. Though certain local portions of these real networks are different from such a lattice in that they cannot distinctively affect the behaviors of other portions of the network. Therefore, a lattice network is used in the experiments of this study. We created a 50 × 50 (2,500-node) undirected lattice network to represent a combined system, where each node represents a subsystem. Then, we randomly initialized the weight of each edge using a Gaussian distribution (1, 0.2) and ensured that there were no negative values. Node betweenness is an important and effective parameter to measure the importance of a node in a network and can describe the load of a node. In these experiments, the load of a single node is the number of shortest paths (i.e., minimum total weight) that pass through it. In this study, we used a fast algorithm based on the Dijkstra Algorithm to calculate all of the shortest paths in a network and the betweenness of each node [14]. To simulate an overload failure, we assumed that a node breaks down when its betweenness is 1 + α times higher than its initial value. When attackers seek to destroy a system, they typically focus on vital subsystems (i.e., central subsystems) or subsystems that tend to experience higher loads that will break down more easily. Thus, to start the cascading failure process, we initially attack the center 4 × 4 nodes and delete them from the network. Then, we recalculate the betweenness of each node that remains functional and delete the nodes whose betweenness is 1 + α times higher than its initial value. We repeat the above process until no node is deleted due to overload (Fig 1).

As shown in Fig 1, failures are generally distant from the center after the initial attack and occur at a constant velocity. However, new failures that occur in one step lie at several different distances. If we want to know how many failures arise at distance 1, 2, 3…, respectively, a constant velocity cannot provide enough information. Thus, we divided the nodes into different groups based on their spatial characteristics and proposed a method of predicting the failure rate of different distances at different times.

### Machine-learning models

We use three machine-learning methods in this study to predict the failure rates of networks at different distances: the feedforward neural network (FNN), the recurrent neural network (RNN) and support vector regression (SVR) [15–17]. These three methods have been proven by different researchers to perform well in nonlinear regression and can achieve high accuracies in prediction problems related to society, economy and technology. The performance of these non-parametric models is strongly related to their architecture, such as the number of hidden layers, the number of hidden nodes, training algorithms and weight adjustments. A well-defined model with an appropriate training algorithm or other optimization methods can produce highly accurate predictions. These non-parametric models are more flexible than parametric models because they can predict data based solely on historical data, while parametric models require certain assumptions to fit different conditions.

**Fig 2:**
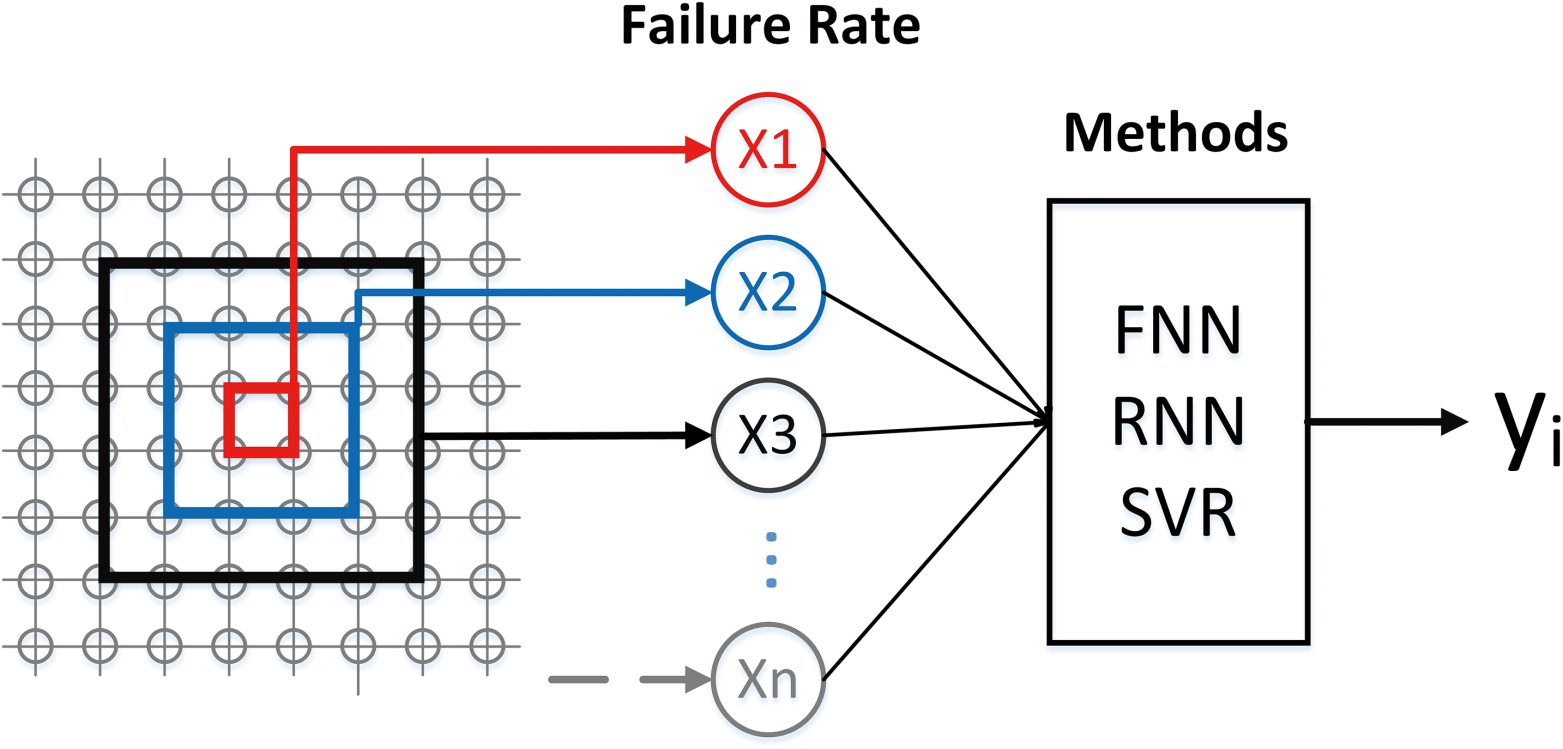
Distance division and model description. Y_i_ is the failure rate of distance i in next step.

In this study, we use distance as the selected spatial feature. Considering that the lattice network is square-shaped, and the nodes that are initially attacked are also square-shaped, we divide different distances by different squares surrounding the central 2×2 nodes; thus, a 50×50 lattice network has 25 different distances. The failure rate of distance d indicates the percentage of failed nodes that lie at this distance away from the central nodes (Fig 2).

For each model, we trained 23 predictors for 23 different distances because distances 1 and 2 do not require prediction. Then, we define each predictor as follows (Fig 2): 25 inputs (i.e., the current failure rates of all distances), and 1 output (i.e., the failure rate of its target distance in next step).

We created 23 predictors for different distances rather than creating a single model that contains 25 inputs and 23 outputs (i.e., the failure rates of 23 distances at a time) because the performance of the model decreases as the number of outputs increases, even though the latter could reduce the computation required in this experiment. A total of 23 outputs make the model unstable and unable to attain even an approximately optimal solution.

**Fig 3:**
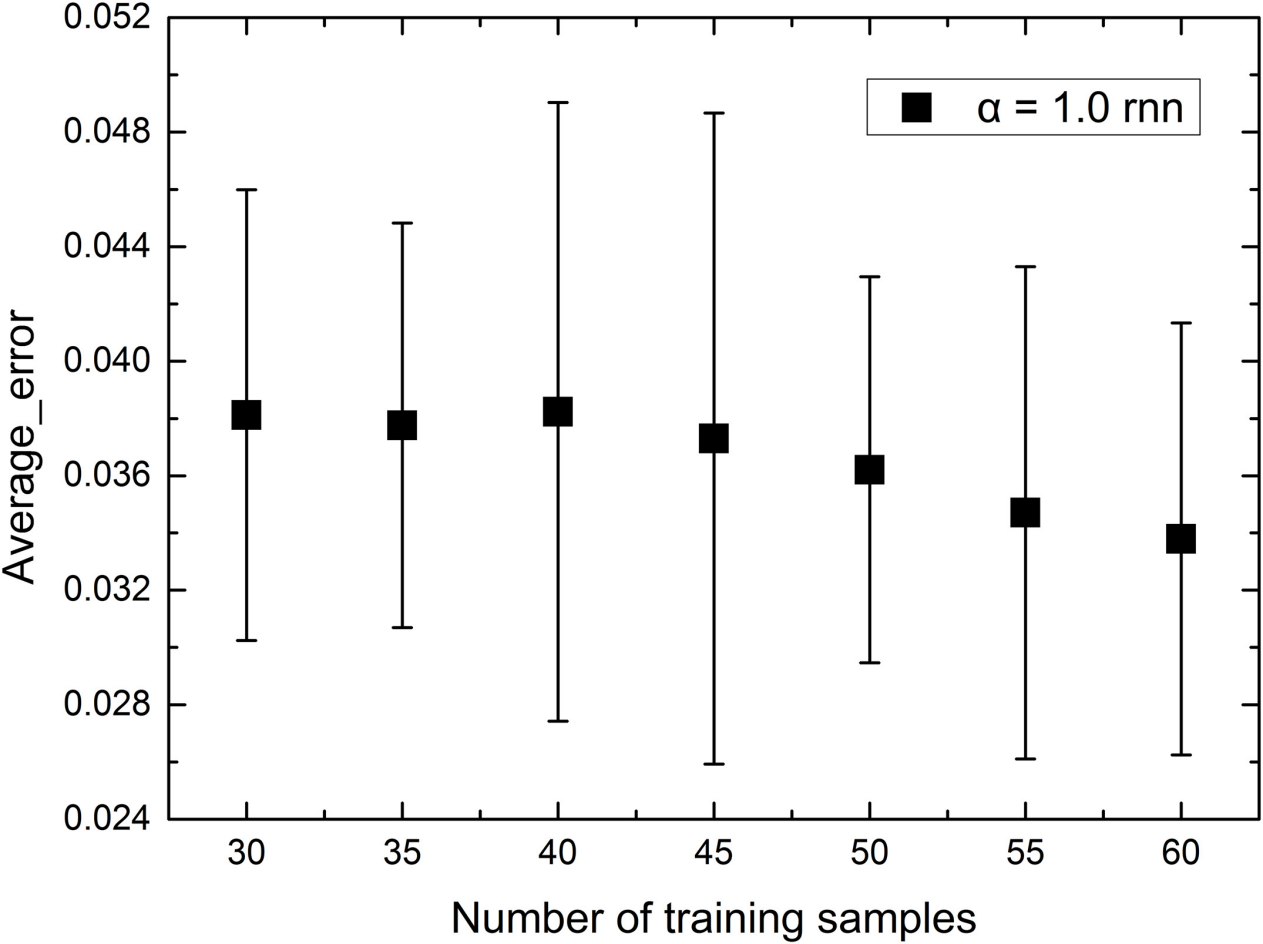
Average errors and deviations of different sizes of training samples.

To acquire the data used for training and testing, we constructed 100 lattice networks of size 50×50 whose edges were initialized by the Gaussian distribution (1, 0.2). Then, with a tolerance α of [0.2, 0.6, 1.0, 1.4, 1.8, 2.2], where a node will fail if its betweenness is 1 + α times higher than its initial value, we calculated the cascading process of the constructed lattice networks that exhibit different edge weight distributions. Thus, for each α, we have 100 groups of cascading failure data. Because different α values present different laws in the cascading failure process, we first attempt to train and test data for a given α. In this study, we performed certain pre-tests to choose the size of the training samples. With α = 1.0, we trained several RNN models with training samples of different sizes that ranged from 30 to 60. Then, we tested the models using the same five groups of data, which were not included in any training dataset. The average errors of each model and its deviation (Fig 3) showed that the average error of the model not markedly declined as the number of training samples increased. However, the model trained with 45 samples became unstable because its deviation was larger than those of the other models. If the unstable models performed well, the model trained by other sample sizes would also perform well. Therefore, we decided to train the proposed models using 45 samples.

**Fig 4:**
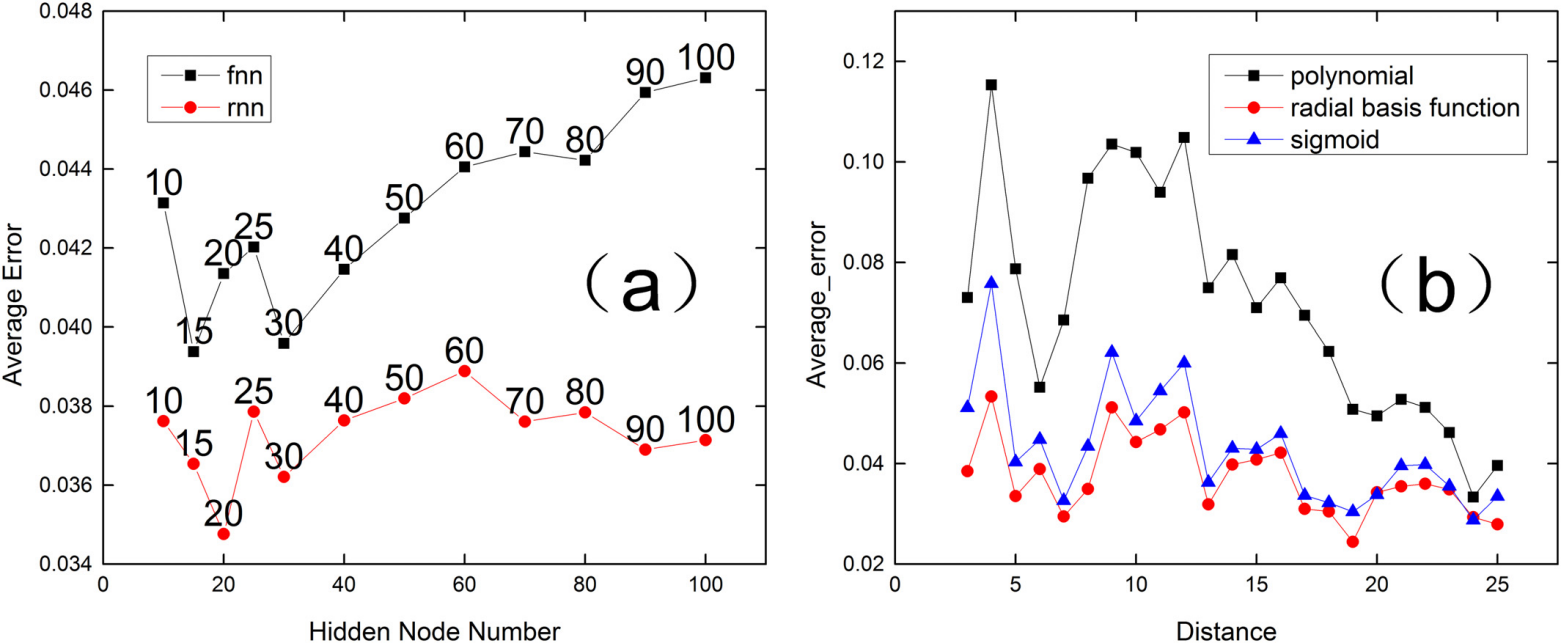
Performances of different structures of each method, with α = 1.0. (a) Average errors of different hidden node numbers for the RNN and FNN models. (b) Average errors of different kernel functions for the SVR model.

For the FNN models, we used the FeedForwardNetwork module of Pybrain [18] to build the proposed FNN model. Each FNN model contains a linear input layer with 25 nodes, a linear output layer with 1 node, and a “Tanh” hidden layer with an undetermined number of nodes. The number of hidden nodes is determined based on experimental results. Different layers are fully connected. For the RNN model, we used a “vanilla” RNN model that was implemented based on the library named Theano. Similar to the FNN model above, each RNN model contains a linear input layer with 25 nodes and a linear output layer with 1 node. The activation of nodes in the hidden layer remains Tanh. Because the FNN and RNN models both require the number of hidden nodes to be chosen, we considered different FNN and RNN models with different numbers of hidden nodes for α = 1.0. Then, we calculated the average errors at different distances (Fig 4). Fig 4a shows that FNN and RNN models with 15–30 hidden nodes exhibit the best performance. With regard to both efficiency and accuracy, FNN models with 15 hidden nodes and RNN models with 20 hidden nodes are chosen for use in the experiments of this study. For the SVR model, we used Libsvm [19], which is the most popular support vector machine (SVM) library when building SVM models. Similarly, we had to select the kernel type of the SVR. Because this is a nonlinear fitting task, we considered three regression kernel functions (polynomial, radial basis function, sigmoid), which were supplied by Libsvm. Based on the result of the average errors shown in Fig 4b, we selected the radial basis function (RBF) as the kernel function in the proposed SVR model.

## Results

### Learning with a given α

For the FNN model, we used a back propagation through time (BPTT) trainer, which is widely used in deep learning, and trained each FNN model using 500 epochs. For the RNN model, we used an efficient Hessian-Free optimizer to train the proposed networks with 500 epochs instead of using the BPTT algorithm in training. Training using the Hessian-Free optimizer is faster than using BPTT, and the accuracy of the two methods is similar. For the SVR model, we did not need to rescale the data because the data were all on the interval [0, 1]. We set the terminator condition such that the cost of the model was less than 0.001.

**Fig 5:**
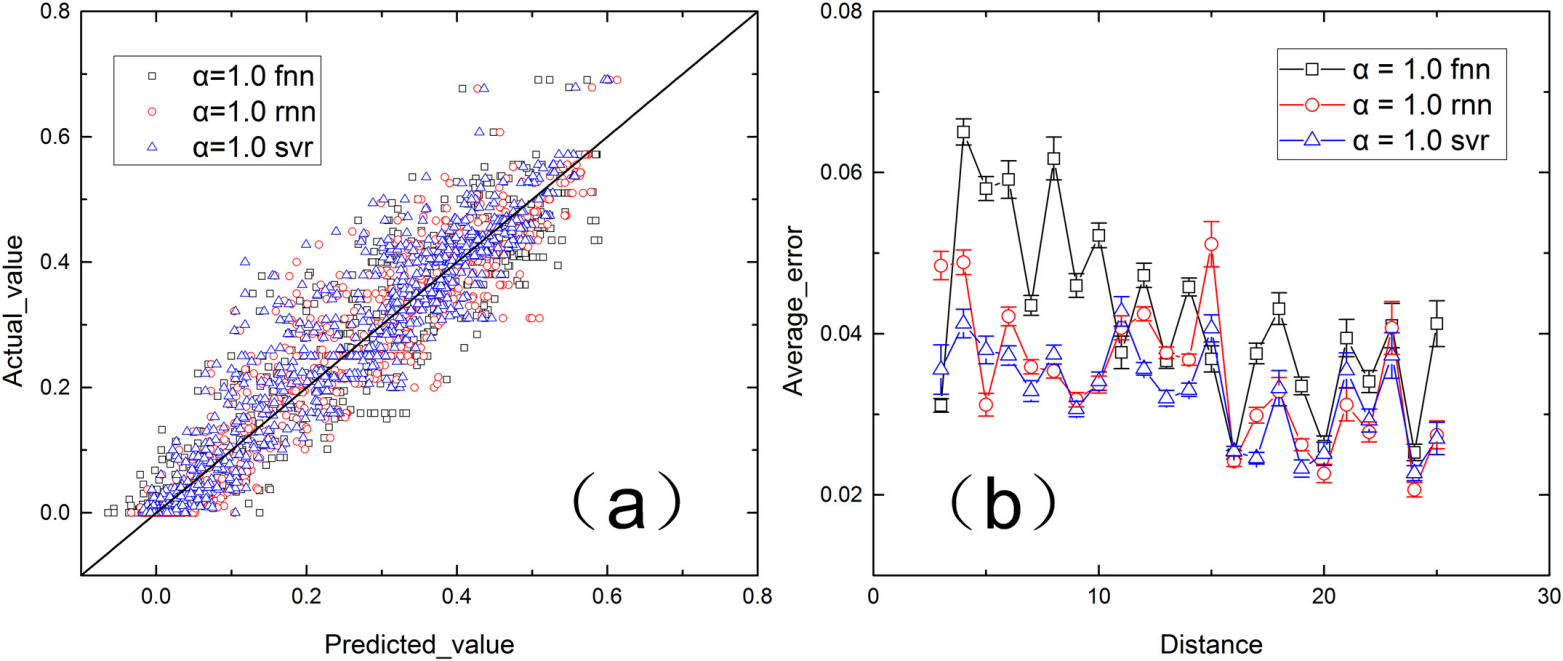
Performances of the three methods, with α = 1.0. (a) Real value vs. predicted value of different models. (b) Average error of each distance.

After training the models, we tested the predictor using the five groups of test data. In this study, only the results of α = 1.0 are shown because the results of different values of α are similar. First, we drew a predicted value-actual value figure (Fig 5a) to show the results of the proposed models. Then, we calculated the average absolute error (|predicted value—real value|/n) and the deviation of each distance produced by the different learning models (Fig 5b).

The nodes in Fig 5a primarily lie near the line y = x, which demonstrates that models trained for a certain tolerance can accurately predict the failure rates of different distances at different times. The average errors of different learning models, which are shown in Fig 5b, do not differ considerably, which demonstrates that the results of different models were near their optimal solutions. Also, the small standard deviation of each distance indicates that the models are all stable. In complex regression problems, the optimal solution cannot be easily obtained within limited computation. After training in several epochs, the cost function fluctuates within a small range or decreases slowly, which indicates that the current solution is near the optimal solution and can be used as the optimal solution. Continuing training to reach the optimal solution would require high computation costs, and obtaining the optimal solution can only improve the performance of the model marginally.

**Fig 6:**
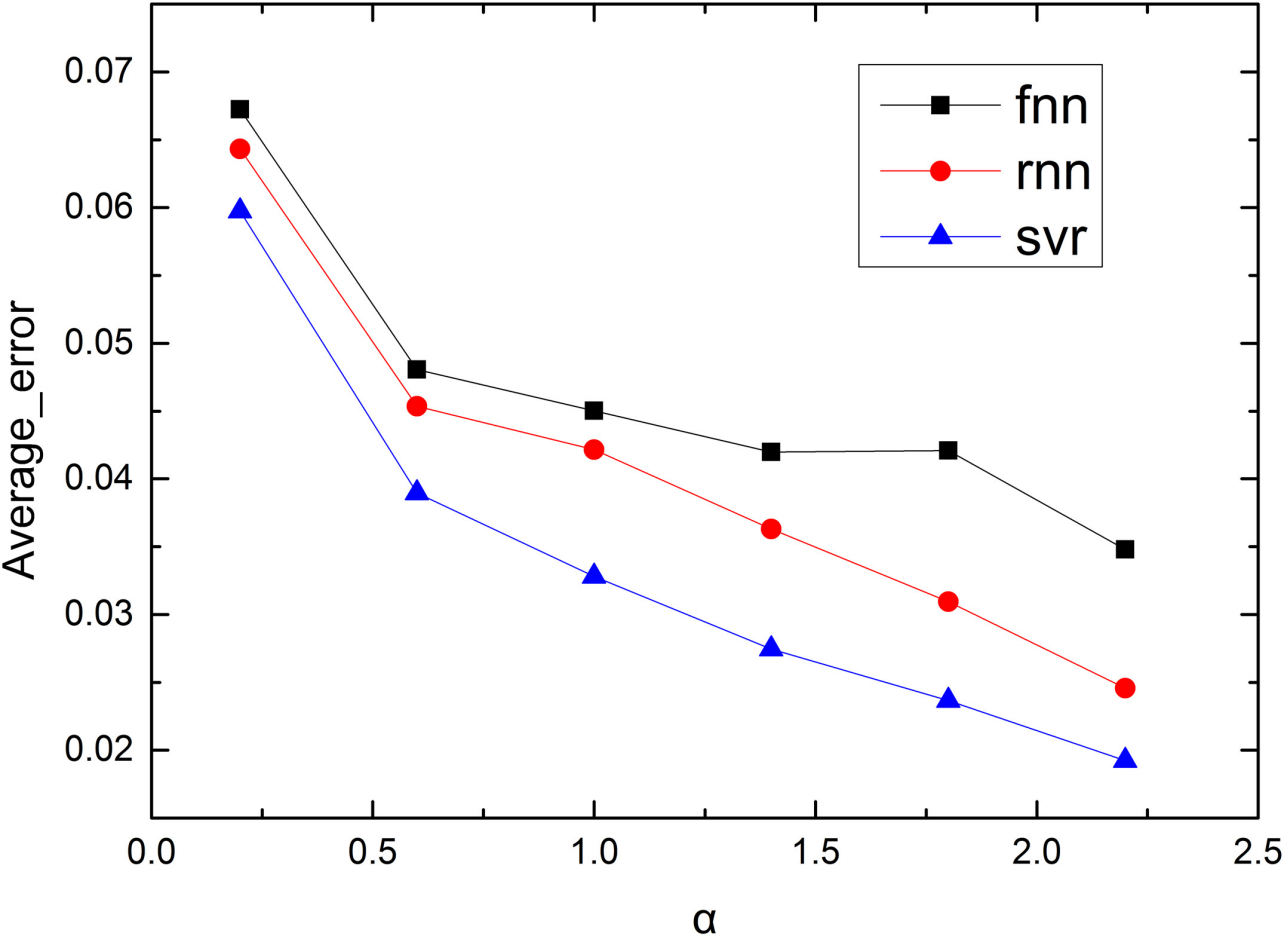
Average error of different distance errors with different values of α.

Because there are 23 predictors for each α, and each predictor has its own error, we calculated the average error of different predictors for each α to fully describe the accuracy of the predictors with different values of α (Fig 6). The average errors of different models are shown to decrease as α increases and show similar trends. The errors decrease as α increases because nodes are sensitive to overload when α is small, and the network changes rapidly with time. Many failures occur in a single step, which increases the randomness of the cascading failure process. The similar decreasing trends of the different models also indicate that each model has reached an approximately optimal solution.

### Learning without a given α

Training models with a given α increases the limitation that if we want to predict failures in a system, we must know the exact value of α and choose the corresponding predictors that have been trained under the same α. In reality, the α of a system is unknown in certain cases, which results in a significant loss when we train predictors considering different tolerances. Thus, a new prediction model that can be used under different circumstances without considering a given α is required. First, to include the information of α into the inputs of a predictor, we added the failure rates of step t-2 to the input to predict the failure fates of step t instead of using the failure rates of step t-1 as the input. Thus, the nodes of the input increase from 25 to 50, and the α of the system is hidden in the inputs, which can be learned by the new models.

**Fig 7:**
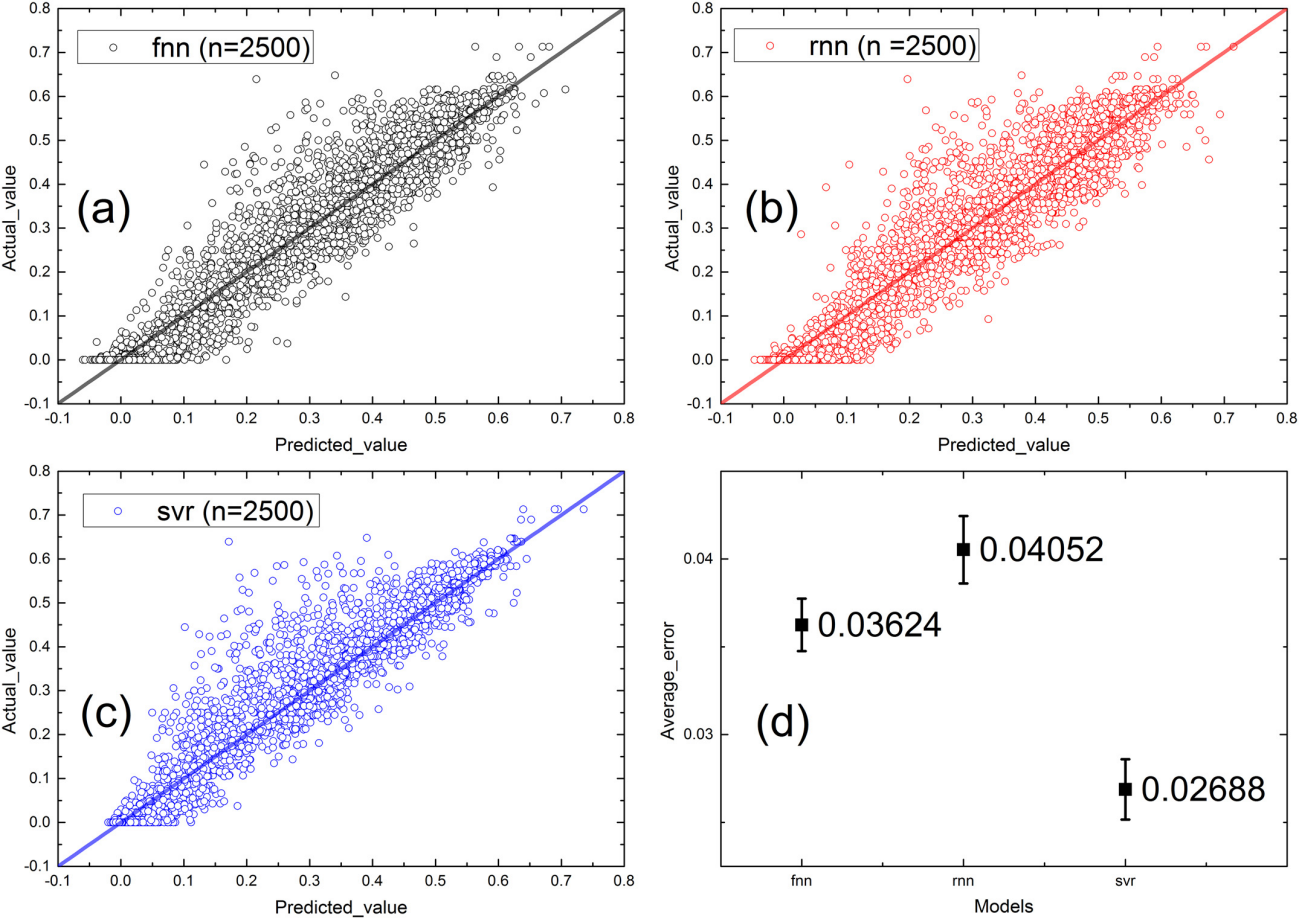
Real value vs. predicted value of different models without considering α, n = 2500. (a) FNN. (b) RNN. (c) SVR. (d) Average error of each model.

Second, to make the results more accurate, we each calculated five groups of cascading failures for α on the interval [0.4, 0.8, 1.2, 1.6, 2.0] and used these groups of data as the testing dataset. Because the training dataset included data from α on the interval [0.2, 0.6, 1.0, 1.4, 1.8, 2.2], which are different from the testing data, we increased the randomness of the α in the proposed experiments. Aside from adding 25 nodes to the input and changing the test dataset, we did not change the other parts of the models and repeated the experiment in a similar manner to that described in the last section. Because the results of all of the predicted values with the corresponding real values cannot be clearly presented in a single figure, we present them in different figures based on the different learning methods. Because the points primarily lie near the line y = x, the new model containing 50 inputs that was used to predict failure rates without knowing α still performs well. The similar results of the three methods demonstrate that each method reached an appropriate optimal solution, and the small deviations signify the stability of the methods (Fig 7).

**Fig 8:**
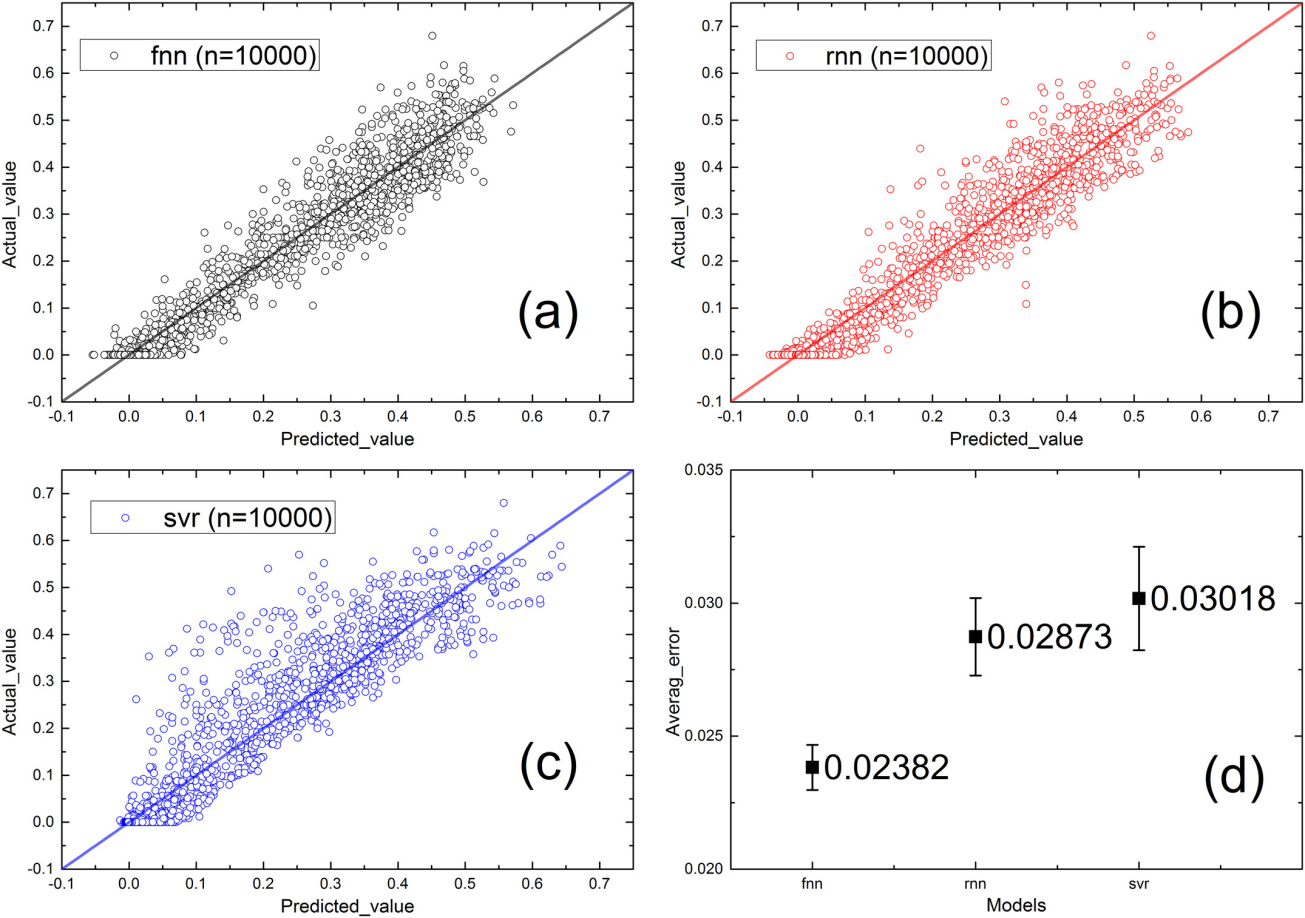
Real value vs. predicted value of different models without considering α, n = 10000. (a) FNN. (b) RNN. (c) SVR. (d) Average error of each model.

We then repeated the proposed experiment on a larger lattice network with a size of 100×100; these results are shown in Fig 8. The three methods still perform well because they each present a small average absolute error and a narrow standard deviation. The SVR model is shown to perform marginally worse than the other two neural networks in this study; however, on the network with a size of 50×50, the SVR model performs better than the other models. This may occur because the neural networks are more complex and perform better at solving problems with multidimensional inputs.

## Discussion

Cascading overload failures occur in large-scale spatial systems and present visible temporal and spatial correlations. Previous studies have considered the failure prediction of a simple target over time, and most studies have investigated the reliability of an entire system or a single component. Because the size of most systems is gradually increasing, it is not possible to concentrate on one or two specific nodes in the network when failures spread. As a result, we seek to determine what is occurring in a group of nodes that share the same spatial characteristics. We proposed a failure rate prediction at different distances using the FNN, RNN and SVR models, and found that these methods can accurately predict failure rates in networks.

First, assuming the α of the system is known, we trained and tested the models under a certain α. The experimental results demonstrated that the proposed prediction model could accurately predict the failure rate with a small error, and the three learning methods all performed well and reached their approximate optimal solutions. However, considering that the α of a system is unknown in certain cases, we changed the proposed models by adding the data of t-2 to the input to include the information of α in the training data. We only trained predictors for different distances without considering α. The results showed that the predictors trained for uncertain α performed well and had a similar average error to that of the predictors trained for given α. However, the model that did not consider α cannot predict the failure rates of the step immediately after the initial attack. When α is known, the model trained for a given α is more powerful and more efficient than models that do not consider α, even though the latter are more convenient.

Forecasting the behavior of every node in a network that is experiencing cascading failures would provide more useful information. However, this is difficult because the behavior of a node is based on many factors, including its status and function, its neighbors’ status and functions, and the relationships between these nodes. In addition to the challenge of choosing an appropriate model to predict a node’s behavior, we must know what type of information and how much information should be input to the model. This topic will be addressed in future work.
